# Assessment of white matter hyperintensity severity using multimodal magnetic resonance imaging

**DOI:** 10.1093/braincomms/fcad279

**Published:** 2023-10-19

**Authors:** Olivier Parent, Aurélie Bussy, Gabriel Allan Devenyi, Alyssa Dai, Manuela Costantino, Stephanie Tullo, Alyssa Salaciak, Saashi Bedford, Sarah Farzin, Marie-Lise Béland, Vanessa Valiquette, Sylvia Villeneuve, Judes Poirier, Christine Lucas Tardif, Mahsa Dadar, the PREVENT-AD Research Group, Angela Tam, Anne Labonté, Alexa Pichet Binette, Anne-Marie Faubert, Axel Mathieu, Cécile Madjar, Charles Edouard Carrier, Christian Dansereau, Christina Kazazian, Claude Lepage, Cynthia Picard, David Maillet, Diane Michaud, Doris Couture, Doris Dea, Claudio Cuello, Alan Barkun, Alan Evans, Blandine Courcot, Christine Tardif, Clément Debacker, Clifford R Jack, David Fontaine, David S Knopman, Gerhard Multhaup, Jamie Near, Jeannie-Marie Leoutsakos, Jean-Robert Maltais, Jason Brandt, Jens Pruessner, John C Morris, John C S Breitner, Judes Poirier, Laksanun Cheewakriengkrai, Lisa-Marie MÃ¼nter, Louis Collins, Mallar Chakravarty, Mark A Sager, Marina Dauar-Tedeschi, Mark Eisenberg, Natasha Rajah, Paul Aisen, Paule-Joanne Toussaint, Pedro Rosa-Neto, Pierre Bellec, Penelope Kostopoulos, Pierre Etienne, Pierre N Tariot, Pierre Orban, Reisa A Sperling, Rick Hoge, Ronald G Thomas, Serge Gauthier, Suzanne Craft, Sylvia Villeneuve, Thomas J Montine, Vasavan Nair, Véronique Bohbot, Vinod Venugopalan, Vladimir Fonov, Yasser Ituria-Medina, Zaven S Khachaturian, Eduard Teigner, Elena Anthal, Elsa Yu, Fabiola Ferdinand, Galina Pogossova, Ginette Mayrand, Guerda Duclair, Guylaine Gagné, Holly Newbold-Fox, Illana Leppert, Isabelle Vallée, Jacob Vogel, Jennifer Tremblay-Mercier, Joanne Frenette, Josée Frappier, Justin Kat, Justin Miron, Karen Wan, Laura Mahar, Leopoldina Carmo, Louise Théroux, Mahsa Dadar, Marianne Dufour, Marie-Elyse Lafaille-Magnan, Melissa Appleby, Mélissa Savard, Miranda Tuwaig, Mirela Petkova, Pierre Rioux, Pierre-FranÃ§ois Meyer, Rana El-Khoury, Renee Gordon, Renuka Giles, Samir Das, Seqian Wang, Shirin Tabrizi, Sulantha Mathotaarachchi, Sylvie Dubuc, Tanya Lee, Thomas Beaudry, Valérie Gervais, Véronique Pagé, Julie Gonneaud, GÃ¼lebru Ayranci, Tharick A Pascoal, René Desautels, Fatiha Benbouhoud, Eunice Farah Saint-Fort, Sander C J Verfaillie, Sarah Farzin, Alyssa Salaciak, Stephanie Tullo, Etienne Vachon-Presseau, Leslie-Ann Daoust, Theresa KÃ¶be, Nathan Spreng, Melissa McSweeney, Nathalie Nilsson, Morteza Pishnamazi, Christophe Bedetti, Louise Hudon, Claudia Greco, Jean-Paul Soucy, M Mallar Chakravarty

**Affiliations:** Cerebral Imaging Centre, Douglas Mental Health University Institute, Montreal, Quebec H4H 1R3, Canada; Integrated Program in Neuroscience, McGill University, Montreal, Quebec H3A 1A1, Canada; Cerebral Imaging Centre, Douglas Mental Health University Institute, Montreal, Quebec H4H 1R3, Canada; Integrated Program in Neuroscience, McGill University, Montreal, Quebec H3A 1A1, Canada; Cerebral Imaging Centre, Douglas Mental Health University Institute, Montreal, Quebec H4H 1R3, Canada; Department of Psychiatry, McGill University, Montreal, Quebec H3A 1A1, Canada; Cerebral Imaging Centre, Douglas Mental Health University Institute, Montreal, Quebec H4H 1R3, Canada; Integrated Program in Neuroscience, McGill University, Montreal, Quebec H3A 1A1, Canada; McConnell Brain Imaging Centre, Montreal Neurological Institute, Montreal, Quebec H3A 2B4, Canada; Cerebral Imaging Centre, Douglas Mental Health University Institute, Montreal, Quebec H4H 1R3, Canada; Cerebral Imaging Centre, Douglas Mental Health University Institute, Montreal, Quebec H4H 1R3, Canada; Integrated Program in Neuroscience, McGill University, Montreal, Quebec H3A 1A1, Canada; Cerebral Imaging Centre, Douglas Mental Health University Institute, Montreal, Quebec H4H 1R3, Canada; Cerebral Imaging Centre, Douglas Mental Health University Institute, Montreal, Quebec H4H 1R3, Canada; Department of Psychiatry, McGill University, Montreal, Quebec H3A 1A1, Canada; Cerebral Imaging Centre, Douglas Mental Health University Institute, Montreal, Quebec H4H 1R3, Canada; Cerebral Imaging Centre, Douglas Mental Health University Institute, Montreal, Quebec H4H 1R3, Canada; Cerebral Imaging Centre, Douglas Mental Health University Institute, Montreal, Quebec H4H 1R3, Canada; Integrated Program in Neuroscience, McGill University, Montreal, Quebec H3A 1A1, Canada; Cerebral Imaging Centre, Douglas Mental Health University Institute, Montreal, Quebec H4H 1R3, Canada; Department of Psychiatry, McGill University, Montreal, Quebec H3A 1A1, Canada; Center for the Studies in the Prevention of Alzheimer's Disease, Douglas Mental Health University Institute, Montreal, Quebec H4H 1R3, Canada; McConnell Brain Imaging Centre, Montreal Neurological Institute, Montreal, Quebec H3A 2B4, Canada; Department of Psychiatry, McGill University, Montreal, Quebec H3A 1A1, Canada; Center for the Studies in the Prevention of Alzheimer's Disease, Douglas Mental Health University Institute, Montreal, Quebec H4H 1R3, Canada; Molecular Neurobiology Unit, Douglas Mental Health University Institute, Montreal, Quebec H4H 1R3, Canada; Department of Medicine, McGill University, Montreal, Quebec H4A 3J1, Canada; McConnell Brain Imaging Centre, Montreal Neurological Institute, Montreal, Quebec H3A 2B4, Canada; Department of Biomedical Engineering, McGill University, Montreal, Quebec H3A 2B4, Canada; Department of Neurology and Neurosurgery, McGill University, Montreal, Quebec H3A 1A1, Canada; Department of Psychiatry, McGill University, Montreal, Quebec H3A 1A1, Canada; Center for the Studies in the Prevention of Alzheimer's Disease, Douglas Mental Health University Institute, Montreal, Quebec H4H 1R3, Canada; Cerebral Imaging Centre, Douglas Mental Health University Institute, Montreal, Quebec H4H 1R3, Canada; Integrated Program in Neuroscience, McGill University, Montreal, Quebec H3A 1A1, Canada; Department of Psychiatry, McGill University, Montreal, Quebec H3A 1A1, Canada; Department of Biomedical Engineering, McGill University, Montreal, Quebec H3A 2B4, Canada

**Keywords:** small vessel disease, dementia, microstructure, relaxometry, cerebrovascular disease

## Abstract

White matter hyperintensities are radiological abnormalities reflecting cerebrovascular dysfunction detectable using MRI. White matter hyperintensities are often present in individuals at the later stages of the lifespan and in prodromal stages in the Alzheimer’s disease spectrum. Tissue alterations underlying white matter hyperintensities may include demyelination, inflammation and oedema, but these are highly variable by neuroanatomical location and between individuals. There is a crucial need to characterize these white matter hyperintensity tissue alterations *in vivo* to improve prognosis and, potentially, treatment outcomes. How different MRI measure(s) of tissue microstructure capture clinically-relevant white matter hyperintensity tissue damage is currently unknown. Here, we compared six MRI signal measures sampled within white matter hyperintensities and their associations with multiple clinically-relevant outcomes, consisting of global and cortical brain morphometry, cognitive function, diagnostic and demographic differences and cardiovascular risk factors. We used cross-sectional data from 118 participants: healthy controls (*n* = 30), individuals at high risk for Alzheimer’s disease due to familial history (*n* = 47), mild cognitive impairment (*n* = 32) and clinical Alzheimer’s disease dementia (*n* = 9). We sampled the median signal within white matter hyperintensities on weighted MRI images [T_1_-weighted (T1w), T_2_-weighted (T2w), T1w/T2w ratio, fluid-attenuated inversion recovery (FLAIR)] as well as the relaxation times from quantitative T1 (qT1) and T2* (qT2*) images. qT2* and fluid-attenuated inversion recovery signals within white matter hyperintensities displayed different age- and disease-related trends compared to normal-appearing white matter signals, suggesting sensitivity to white matter hyperintensity-specific tissue deterioration. Further, white matter hyperintensity qT2*, particularly in periventricular and occipital white matter regions, was consistently associated with all types of clinically-relevant outcomes in both univariate and multivariate analyses and across two parcellation schemes. qT1 and fluid-attenuated inversion recovery measures showed consistent clinical relationships in multivariate but not univariate analyses, while T1w, T2w and T1w/T2w ratio measures were not consistently associated with clinical variables. We observed that the qT2* signal was sensitive to clinically-relevant microstructural tissue alterations specific to white matter hyperintensities. Our results suggest that combining volumetric and signal measures of white matter hyperintensity should be considered to fully characterize the severity of white matter hyperintensities *in vivo*. These findings may have implications in determining the reversibility of white matter hyperintensities and the potential efficacy of cardio- and cerebrovascular treatments.

## Teaser

Parent *et al*. have used multimodal MRI to measure tissue damage within white matter hyperintensities (radiological indicators of vascular dysfunction prevalent in aging and Alzheimer’s disease) and concluded that T2* is a microstructural measure sensitive to clinically-meaningful variations in white matter hyperintensity tissue damage.

## Graphical Abstract

**Graphical Abstract:**
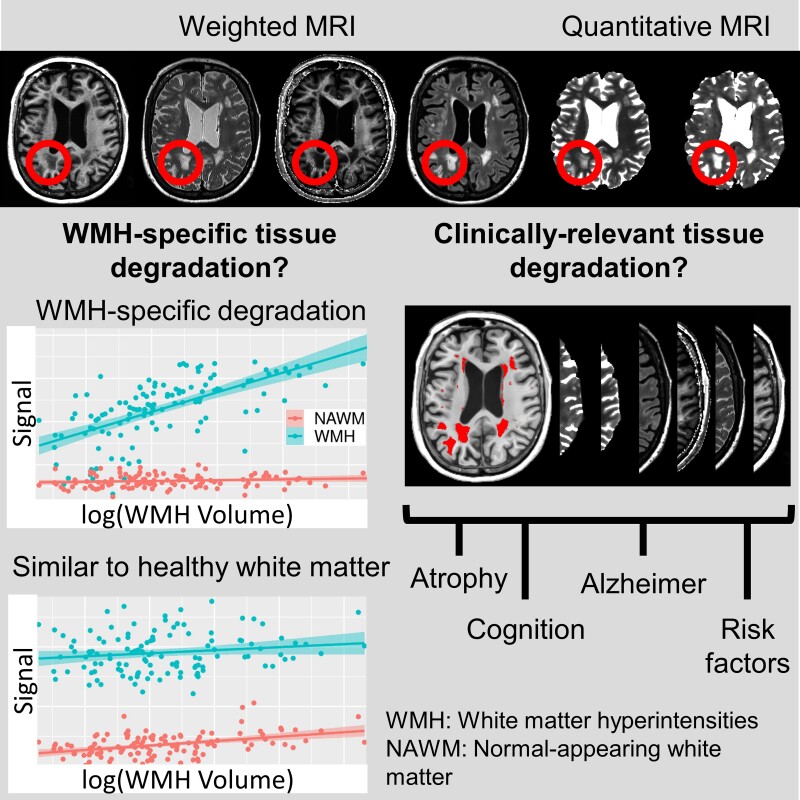

## Introduction

White matter hyperintensities (WMHs) are areas of higher MRI signal within white matter on T_2_-weighted (T2w) and fluid-attenuated inversion recovery (FLAIR) images. WMHs are commonly detected in the elderly and are considered to be markers of small vessel disease. Cerebrovascular disease burden, often measured with the total WMH volume, is increasingly recognized to play an important role in cognitive manifestations of Alzheimer’s disease,^1-3^ with abnormalities in WMH volume detected up to 20 years before Alzheimer’s disease diagnosis.^4^ Increased WMH volumes have also been consistently associated with impaired cognition,^5-7^ cortical atrophy^8-11^ and cardiovascular risk factors^12-14^ in otherwise cognitively normal individuals.

Previous studies have only examined the extent (i.e. volume) of WMHs in relation to adverse outcomes, but not the severity of underlying tissue alterations. Importantly, *ex vivo* histological examinations of microstructural alterations within WMHs report heterogeneous tissue alterations,^15,16^ with demyelination, axonal loss and inflammation being present at various degrees or even absent.^17-20^ There is therefore a crucial need to better assess the severity of WMH microstructural alterations *in vivo*. MRI signals from different types of MRI images may serve as a means to assess this severity. While studies have used T1w, T2w, T1w/T2w ratio (a non-specific proxy for myelin concentration)^21^ and FLAIR signal to index microstructure,^22-24^ these measures generally lack neurobiological specificity.^25^ We hypothesize that novel quantitative MRI acquisitions directly measuring T1 and T2* relaxation times are better suited to characterize WMH tissue damage and may offer a more nuanced description of WMH severity above and beyond overall volume. In white matter, quantitative T1 relaxation time (qT1) is influenced by myelin to a larger extent than iron,^26^ while quantitative T2* relaxation time (qT2*) has been linked to iron, myelin and fibre orientation.^26,27^ As such, these metrics are sensitive to pathologically-relevant biological properties in the context of WMHs. However, it is unknown which MRI measure(s) of tissue microstructure would capture clinically-relevant WMH tissue damage.

In this study, we compared the clinical associations of MRI signal measures of WMH. Leveraging the high variability of WMH severity, neurodegeneration and cognitive functioning in the Alzheimer’s disease spectrum, we measured six different MRI signals within WMHs and the normal-appearing white matter (NAWM) in a cross-sectional sample including cognitively healthy elderly, participants at high risk of Alzheimer’s disease due to familial history, participants with mild cognitive impairment and a few participants with clinically-diagnosed Alzheimer’s disease totalling 118 subjects. We first assessed if the WMH signal trends simply represented deterioration of the global white matter, or if these trends were specific to WMHs (i.e. different than trends in the NAWM). We then related WMH measures to multiple types of clinical variables (cortical and global atrophy, cognition, demographic and group differences and cardiovascular risk factors), with the rationale that signal measures sensitive to clinically-meaningful variations in underlying WMH tissue alterations would be related to adverse neurobiological and clinical outcomes.

## Materials and methods

### Participants

**Figure 1:**
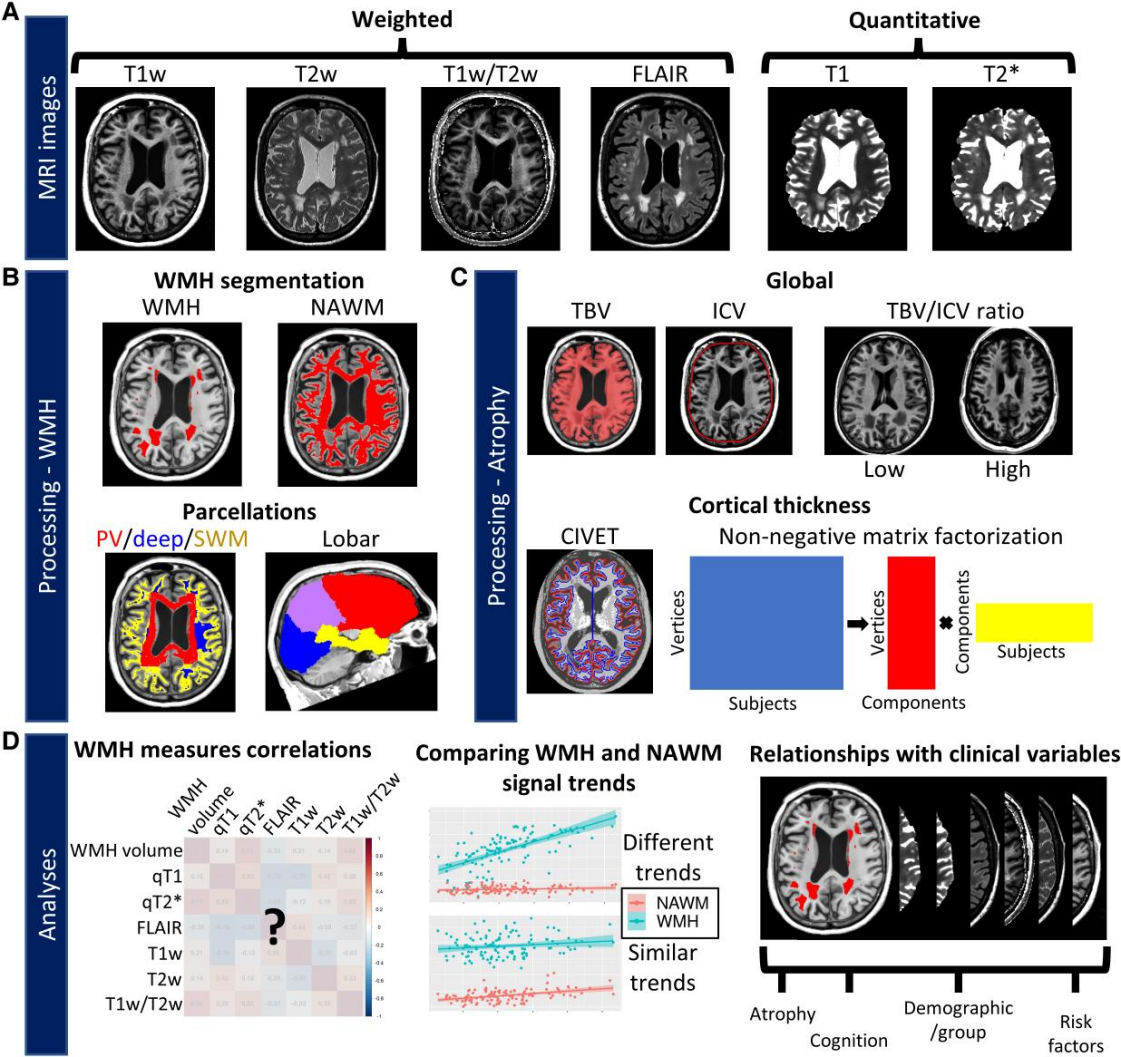
**Workflow of MRI acquisitions, processing and analyses.** (**A**) Six types of MR images were acquired and processed. Figures are from a 74-year-old female participant with Alzheimer’s disease and high WMH volume. (**B**) *Top*: white matter tissue was separated into WMH and NAWM. *Bottom*: both tissue types were parcellated with a periventricular (PV)/deep/superficial white matter (SWM) and lobar parcellation. Subject-wise median signal was sampled within each subregion and MRI image. (**C**) Atrophy measures included global [total brain volume (TBV), intracranial brain volume (ICV), TBV/ICV ratio] (*top*) and cortical thickness measures (*bottom*). The dimensionality of the vertex-wise cortical thickness data was reduced by deriving a data-driven parcellation using non-negative matrix factorization (decomposition process is shown). (**D**) Three types of analyses were performed: (i) correlations of WMH signal measures between themselves; (ii) comparing WMH and NAWM signal age- and disease-related trends; and (iii) assessing the relationships of WMH measures with clinically-relevant variables (atrophy, cognition, clinical group, cardiovascular risk factors) using univariate and multivariate analyses.

Our methodology is outlined in Fig. 1. The data were acquired as part of the Alzheimer’s Disease Biomarkers (ADB)^22,28-30^ and PREVENT-AD^31,32^ cross-sectional cohorts, recruited between 2016 and 2019. Signed informed consent from all participants was obtained, and the research protocols were approved by the Research Ethics Board of the Douglas Mental Health University Institute, Montreal, Canada. Summary statistics of demographic and cognitive variables before and after quality control (QC) procedures (see Supplementary Methods 1) are detailed in Table 1 (both cohorts combined). ANOVAs and chi-squared tests were used to assess differences in the samples before (*n* = 253) and after (*n* = 118) QC for continuous and categorical variables respectively. No significant differences were observed at the *P* < 0.05 level. A Gantt chart showing the number of exclusions at each QC step is available in Supplementary Fig. 1. Of note, approximately half of the exclusions are based on missing MRI modalities, with another ∼30% based on raw MRI QC, and do not reflect failure of the image processing methods used.

**Table 1:** Summary statistics of demographic and cognitive variables before and after quality control (QC) procedures

	Before quality control		After quality control		
Variable	Statistic	Missing values	Statistic	Missing values	*P*-values
Total *n*	253		118		
Group (HC, FAMHX, MCI, AD)	82, 78, 63, 30	0	30, 47, 32, 9	0	*P* = 0.1902
Age	68.0 ± 6.69 (54–91)	1	66.6 ± 6.33 (54–83)	0	*P* = 0.0531
Sex (male, female)	96, 157	0	42, 76	0	*P* = 0.7481
BMI	26.5 ± 4.68	1	26.1 ± 4.26	0	*P* = 0.4235
Years of education	15.0 ± 8.08	5	15.8 ± 3.96	0	*P* = 0.3187
Number of APOE-ɛ4 alleles (0,1,2)	156, 87, 10	0	75, 37, 6	0	*P* = 0.7761
AD8	1.2 ± 1.76	11	1.1 ± 1.66	2	*P* = 0.8080
MoCA	24.7 ± 4.42	5	25.1 ± 3.94	1	*P* = 0.4015
RBANS attention	98.9 ± 15.76	30	100.3 ± 14.06	10	*P* = 0.6428
RBANS delayed memory	91.92 ± 18.19	33	91.8 ± 17.71	10	*P* = 0.5625
RBANS immediate memory	93.9 ± 17.55	31	94.6 ± 16.79	10	*P* = 0.8791
RBANS visuospatial memory	95.1 ± 15.64	31	94.9 ± 15.74	10	*P* = 0.6226
RBANS language	97.1 ± 12.55	29	97.6 ± 14.35	9	*P* = 0.8546
RBANS total	93.8 ± 14.68	33	94.4 ± 14.63	10	*P* = 0.7784
Alcohol (yes, no)	204, 41	8	103, 12	3	*P* = 0.1335
Diabetes (yes, no)	17, 227	9	8, 107	3	*P* = 1
High BP (yes, no)	76, 169	8	31, 84	3	*P* = 0.4198
High cholesterol (yes, no)	86, 158	9	38, 77	3	*P* = 0.6501
Smoking (yes, no)	17, 227	9	7, 108	3	*P* = 0.8842

Mean ± standard deviations are shown for continuous variables, frequencies for categorical variables, and the age range is displayed. The number of missing values for each variable was computed. The column of missing values after QC also refers to the number of computationally imputed observations included in the analyses. ANOVAs and chi-squared tests were used to assess differences in the samples before and after QC for continuous and categorical variables respectively. *P*-values of those tests are shown. QC, quality control; HC, healthy controls; FAMHX, familial history of Alzheimer’s disease; MCI, mild cognitive impairment; AD, Alzheimer’s disease dementia; BMI, body mass index; MoCA, Montreal Cognitive Assessment; RBANS, Repeatable Battery for the Assessment of Neuropsychological Status; BP, blood pressure.

### Data acquisition

#### Demographics, cognition and cardiovascular risk factors

Participants were recruited across the Alzheimer’s disease spectrum in four clinically-different groups: healthy controls (HC), high risk due to familial history of Alzheimer’s disease (FAMHX), mild cognitive impairment (MCI) and Alzheimer’s disease dementia. Participants with MCI or Alzheimer’s disease were referred to this study after diagnosis by the clinical team at the McGill Centre for Studies in Aging in Montreal, Canada. FAMHX participants were recruited by the PREVENT-AD group, had a Clinical Dementia Rating of 0 and had at least one parent diagnosed with Alzheimer’s disease. HC participants were recruited through advertisements in local newspapers targeted towards aging populations, Facebook and Kijiji posts. Exclusion criteria included psychiatric and intellectual disorders, brain damage and concussion, current use of psychoactive substances and contraindications to MRI.

Demographic variables included the body mass index (BMI) and the number of APOE-ɛ4 alleles calculated with the PCR method^33^ using the Pyrosequencing protocol recommended by the manufacturer. Cognitive assessments included the AD8,^34^ the Montreal Cognitive Assessment (MoCA)^35^ and the Repeatable Battery for the Assessment of Neuropsychological Status (RBANS).^36^ We acquired the self-reported history of alcohol consumption, diabetes, high blood pressure (BP), high cholesterol and smoking in a binary format (yes or no).

Missing values for demographic, cognitive and risk factor variables (see Table 1) were imputed with Random Forest imputation using the missForest version 1.5 package in R^37^ on the complete sample before exclusions based on MRI quality control.

#### MRI acquisition

Identical MRI sequences were acquired for both cohorts (ADB and PREVENT-AD) on a Siemens Trio 3T scanner using a 32-channel head coil at the Cerebral Imaging Center, associated with the Douglas Research Center in Montreal, Canada. Each resulting image for one subject is visualized in Fig. 1A. Acquisition parameters of MRI protocols, including T1w, T2w, FLAIR, quantitative T1 (from an MP2RAGE sequence) and T2* (from a 12-echo GRE sequence) images are detailed in Supplementary 2. All images are 1 mm isotropic or of higher resolution.

### Image processing

Brain-derived measures from each MRI sequence are detailed in Supplementary Methods 3.

#### Global atrophy measures

Prior to brain segmentation, we preprocessed the T1w images with minc-bpipe (https://github.com/CobraLab/minc-bpipe-library), which performed N4 field inhomogeneity correction,^38^ cropping of the neck region and brain mask extraction using the BEaST non-local segmentation technique.^39^ Using the preprocessed T1w images, broad tissue types were segmented with the MINC Classify tool (https://github.com/BIC-MNI/classify). Total brain volume (TBV) was calculated as the total volume of grey and white matter in mm^3^. Intracranial brain volume (ICV) was calculated as the determinant of the transformation (i.e. a volume scaling) of a skull-to-skull registration of the T1w image to the MNI ICBM152 template. We further combined these two metrics by calculating the ratio of TBV to ICV (TBV/ICV ratio), representing a global measure proportional to the emptiness inside the skull, and thus representing global atrophy (Fig. 1C).

#### Cortical thickness

We used the CIVET 2.1.0 pipeline to generate cortical surfaces from the preprocessed T1w images (Fig. 1C).^40,41^ Cortical thickness was estimated at each vertex as the Laplacian distance between the grey–white matter boundary surface and the pial surface. Values were then resampled into MNI space and surface smoothed with a 20 mm full-width half-max heat kernel.^42^

To reduce the dimensionality of cortical thickness data, we derived a data-driven parcellation using orthogonal projective non-negative matrix factorization (NMF),^43-45^ further detailed in Supplementary Methods 4. Briefly, this method identifies covariance patterns by deconstructing an input matrix of vertices by subjects into two matrices: (i) vertices by components (representing the spatial parcellation); and (ii) components by subjects (proportional to the cortical thickness of every subject inside each component) (Fig. 1C). The second matrix is used as the subject-wise measure of cortical thickness. The number of components is determined by analysing the stability and accuracy of the reconstruction across different component granularities. We chose to use eight components since the stability plateaued at six components, while the accuracy increased substantially up to eight components.

#### T1w/T2w ratio processing

The T1w/T2w ratio developed by Glasser *et al*.^21^ has been proposed as being more myelin-sensitive than either contrast alone. To generate the T1w/T2w ratio images, we first downsampled the T2w images from 0.64 to 1 mm isotropic and rigidly registered the T2w images to the subject-specific T1w images (to have matched resolution and space). We then divided the raw T1w images by the matched T2w images, as in Tullo *et al.*^30^

#### WMH segmentation

Before WMH segmentation, non-linear registration of the ADNI template to the subject-specific T1w image was performed with the Advanced Normalization Tools (ANTs) toolbox.^46^ WMHs were segmented using a validated and automated random forest classifier.^47,48^ We used the preprocessed T1w and T2w images as inputs. Despite being classically used for WMH segmentation, FLAIR images were not included in our WMH segmentation processing because the contrast of grey to white matter of our FLAIR images was different to the contrast of the training data, which led to a higher rate of false positive WMH segmentations near the cortical grey matter. As a result, it is possible that less severe lesions were under-segmented. NAWM masks were created by removing the WMH mask dilated by 2 mm from the global white matter mask (Fig. 1B).

#### White matter parcellations

For the main analyses, WMHs and NAWM were parcellated into four regions: global, periventricular (PV), deep and superficial white matter (SWM). While the classical parcellation of WMH only segregates PV and deep white matter regions, we further differentiated WMHs located in the SWM (Fig. 1B). The PV mask was obtained by dilating a mask of the ventricles by 8 mm, similarly to other studies.^49-52^ The SWM mask was obtained by dilating the cortical grey matter mask by 1 mm. In cases where WMHs were in both PV and SWM masks, the WMH was classified as PV. The deep white matter mask contained the remaining white matter voxels. For the supplementary analyses, WMHs and NAWM were parcellated into five regions: global, frontal, parietal, temporal and occipital. WMH lobar localization has been shown to differentially relate to cognition and dementia.^53^ To estimate lobe-specific WMH measures, we used non-linear registration to map the Hammers atlas^54-56^ from ADNI to native T1w space (Fig. 1B).

### WMH measures

In each white matter region (i.e. global and parcellated), we derived the WMH volumes as well as six WMH signal measures: T1w, T2w, T1w/T2w ratio, FLAIR, qT1 qT2*. The WMH volume was calculated in mm^3^, divided by TBV, and log-transformed, as recommended in previous studies.^6^ Of note, WMH volumes are highly correlated with visual scales like the Fazekas scale, which requires the input of a skilled manual rater.^57^ The use of quantitative volumetric measures offers a more accurate and continuous measure of WMH burden. Signal measures were derived by calculating the median signal in each region for each MRI contrast. Median values were chosen in favour of mean values to limit the impact of partial volume effects and outliers on the signal measures. These can be particularly impactful in periventricular regions where signal values partly sampled in the CSF would be important outliers and thus bias the mean signal measures. For quantitative images, we sampled the raw intensities. For qualitative images, we sampled intensities on the bias-field corrected images that we further normalized by dividing by the image-specific median intensity in the genu of the corpus callosum (defined by a manually segmented mask registered to native space), a region where no WMHs were observed in our sample, to limit non-biological sources of between-subject intensity differences. Further, raw intensities in the genu of the corpus callosum did not correlate with WMH volumes for T1w (*r* = −0.17, *P* = 0.062), T2w (*r* = −0.06, *P* = 0.550) and FLAIR (*r* = −0.04, *P* = 0.699) but did correlate significantly for T1w/T2w ratio (*r* = −0.21, *P* = 0.017). This could underestimate T1w/T2w ratio sensitivity to disease processes.

A low number of subjects had less than five WMH voxels in some white matter regions (12 subjects without deep WMHs, and 2 subjects without SWM WMHs). Instead of removing those subjects, which would bias our sample towards subjects with more advanced pathology, we imputed WMH volume values by log-transforming 1/TBV since it is impossible to divide 0, and WMH signal values by the NAWM signal value in the same region for each MRI image (e.g. qT1 in deep WMH was replaced by qT1 in deep NAWM).

In total, for the PV/deep/SWM parcellation, we included 28 WMH measures (7 volume and signal measures ∗ 4 regions), while for the lobar parcellation, we included 35 WMH measures (7 volume and signal measures ∗ 5 regions).

The MRI data were also manually checked for lacunes. Only one subject presented with a lacune, which did not impact the WMH segmentation, hence the WMH signal and volume measures of that subject are not impacted.

### Statistical analysis

All univariate analyses were performed with R/3.5.1. The whole sample, with all clinical groups combined, was analysed together in order to leverage the full variability of WMH severity, neurodegeneration and cognitive functioning across the Alzheimer’s disease spectrum, resulting in higher statistical power to detect clinical associations. Furthermore, previous research has reported within-group associations between WMH volume and cognitive decline for cognitively healthy individuals, people with mild cognitive impairment and people with Alzheimer’s disease.^6^ While these studies did not investigate WMH signal measures, they provide confidence that WMH associations should not be driven by a group confound.

First, to determine if the WMH signal measures were redundant or if they were each sensitive to different sources of microstructural variations, we computed cross-correlations matrices (*P* < 0.01 threshold) between the WMH characteristics in a within-region between-measure fashion, and in a between-region within-measure fashion (Fig. 1D).

Second, we sought to assess if the WMH signal trends simply represented deterioration of the global white matter, or if these trends were specific to the lesions. We thus calculated the divergence of slopes between WMH and NAWM signal relative to age and WMH volume, which we interpreted as indicators of time and vascular burden, respectively. We used linear models with an interaction term (Equations 1 and 2) and thresholded at the *P* < 0.01 level.


(1) Signal∼Age:Whitemattertype

(2) Signal∼WMHvolume:Whitemattertype

Third, to assess the clinical relevance of the microstructural variation captured by the different WMH signal measures, we used linear models to individually relate WMH measures to four types of clinical variables: cortical and global atrophy, cognition, demographic (including clinical groups) and cardiovascular risk factors (Fig. 1D), while covarying for age, sex and years of education. We used multiple linear regression for continuous clinical variables of interest (e.g. atrophy) and ANCOVA for categorical variables of interest (e.g. group differences). As such, the group variable is treated as a categorical variable and not an ordinal variable. We further assessed the added predictive value above the WMH volume for each WMH signal measure by additionally covarying for the region-specific WMH volume (e.g. for PV qT2*, we added PV WMH volume as a covariate). All continuous variables were *Z*-scored before analysis to obtain standardized beta coefficients. We then examined the *P*-values of the clinical term thresholded at *P* < 0.01. We further corrected *P*-values within the relationship matrix with false discovery rate (FDR) correction.^58^ Relationships that survived FDR correction at the 0.1 level were reported. For all significant group effects, pairwise differences were investigated *post hoc* with Tukey contrasts. The standardized beta coefficients, 95% confidence intervals, *P*-values and FDR-corrected *P*-values for all univariate relationships are available on our GitHub (https://github.com/CoBrALab/WMH_Signal_AD_OParent_2023).

Fourth, in order to investigate how signal measures of WMH are related to demographics and cognition in conjunction with traditional brain markers (i.e. cortical thickness and WMH volume), we used the multivariate technique partial least squares correlation (PLSC), detailed in Supplementary Methods 5 and in Supplementary Fig. 2. Briefly, we used behavioural PLSC with the Python/3.9.7 package pyls/0.01 (https://github.com/rmarkello/pyls), which performed singular value decomposition on a correlation matrix that relates each brain variable to each cognitive and demographic variable.^59-62^ This results in latent variables (LVs) representing linear combinations of cognitive and demographic variables that maximally covary with linear combinations of brain variables. We inverted the directionality of LVs when appropriate for ease of interpretation. Of note, diabetes and smoking history variables were discarded from this analysis since they did not have the required level of variance for PLSC.

### Role of the funding sources

Funding sources had no role in the study design, data collection, analysis, interpretation or writing of the manuscript.

## Results

### White matter parcellations

Results of white matter parcellations are shown in Supplementary Fig. 3. In the PV/deep/SWM parcellation, the vast majority of WMH voxels were classified as PV (86%), relatively few were classified as SWM (11%) and a very small fraction were classified as deep (2%). Importantly, the median number of voxels included in deep WMHs was only 20, potentially rendering the calculation of the median signal inside these regions less reliable and more influenced by outliers. In the lobar parcellation of WMHs (Supplementary Fig. 3B), the most affected region was frontal (58%), followed by parietal (17%), temporal (13%) and occipital (8%).

### Spatially varying and moderate correlations between WMH measures

**Figure 2:**
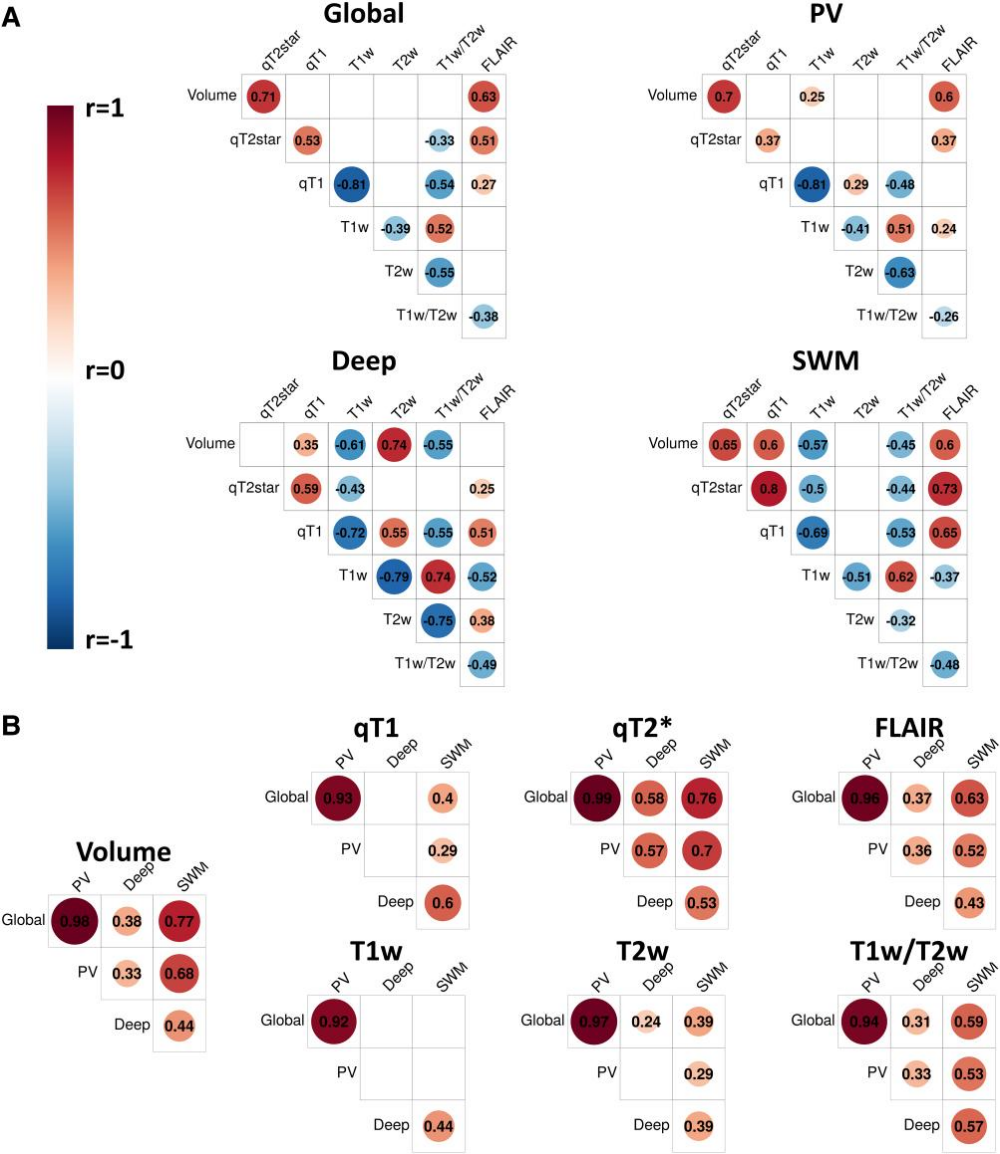
**Correlations of WMH measures.** (**A**) Within-region between-measure correlations of all WMH characteristics (volume and signal measures). (**B**) Between-region within-measure correlations. Circles are proportional to the amplitude of the Pearson’s correlation coefficients, which are also indicated. Warmer colours indicate positive correlations, and colder colours indicate negative correlations. Only significant correlations at *P* < 0.01 are displayed (*n* = 118).

The correlations of WMH measures were analysed within-region between-measure (Fig. 2A) and between-region within-measure (Fig. 2B) with correlations thresholded at *P* < 0.01. Global and PV between-measure relationships were highly similar and generally showed low to moderate correlations (*r* < 0.6) with some exceptions of high correlations (i.e. WMH volume with qT2* and FLAIR; qT1 with T1w). Compared to PV regions, SWM had higher correlations between qT2*, qT1 and FLAIR, while in deep white matter, correlations were higher between WMH volume, T1w, T2w and T1w/T2w ratio.

Subsequently, between-region within-measure relationships were analysed (Fig. 2B). For all WMH characteristics (volume and signal measures), global and PV regions were very highly correlated (*r* > 0.9). For WMH volume, qT2* and FLAIR measures, correlations were highest between global, PV and SWM regions, and deep regions showed lower correlations with other regions. An inverse pattern was observed for qT1 and T1w showing higher relationships between deep and SWM regions compared to other region pairs. T2w and T1w/T2w ratio measures showed higher correlations between SWM and other regions.

### qT2* and FLAIR are sensitive to WMH-specific tissue degradation

**Figure 3:**
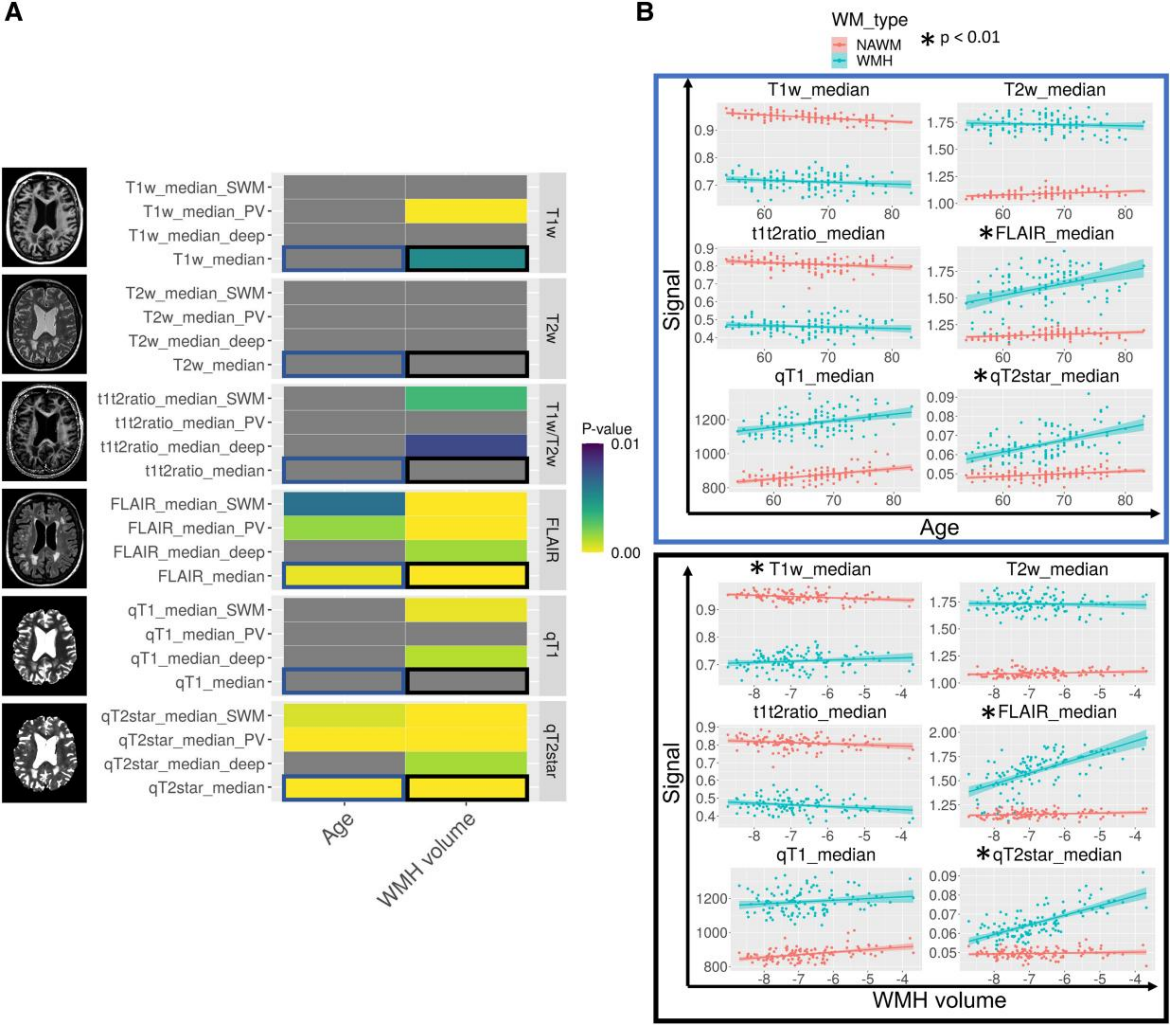
**Comparing signal trends between WMH and NAWM.** (**A**) On the *y*-axis, all WMH signal measures (global and parcellated) are grouped by image type. *P*-values for the interaction term (modelled with linear regression) between either age (*left*) and WMH volume (*right*) with white matter type thresholded at *P* < 0.01 are colour coded, and non-significant associations are in grey. Yellow colours indicate lower *P*-values, and blue colours indicate higher *P*-values. Blue squares indicate relationships for the age term that are visualized, and black squares indicate relationships for the WMH volume term that are visualized. (**B**) Graphical visualization of NAWM (red) and WMH (blue) signal trends in global white matter with age (*top*) and WMH volume (*bottom*) for each signal type. Significantly different white matter trends at the *P* < 0.01 level are indicated with a black star (*n* = 118).

We investigated if the WMH signal trends simply represented deterioration of the global white matter, or if these trends were specific to WMHs. We calculated the divergence of the slopes between WMH and NAWM relative to age and WMH volume (Fig. 3A). Results showed significantly different age- and disease-related signal trends between WMH and NAWM for FLAIR and qT2* in every region except deep white matter. For FLAIR and qT2*, WMH signal increases with age and WMH volume, while NAWM signal remains relatively constant (Fig. 3B). For other measures, there is a large baseline difference between NAWM and WMH but signal trends are highly similar. All significant effects at *P* < 0.01 also survived FDR correction at the 0.05 level.

### Consistent univariate relationships between WMH qT2* signal and clinically-relevant variables

**Figure 4:**
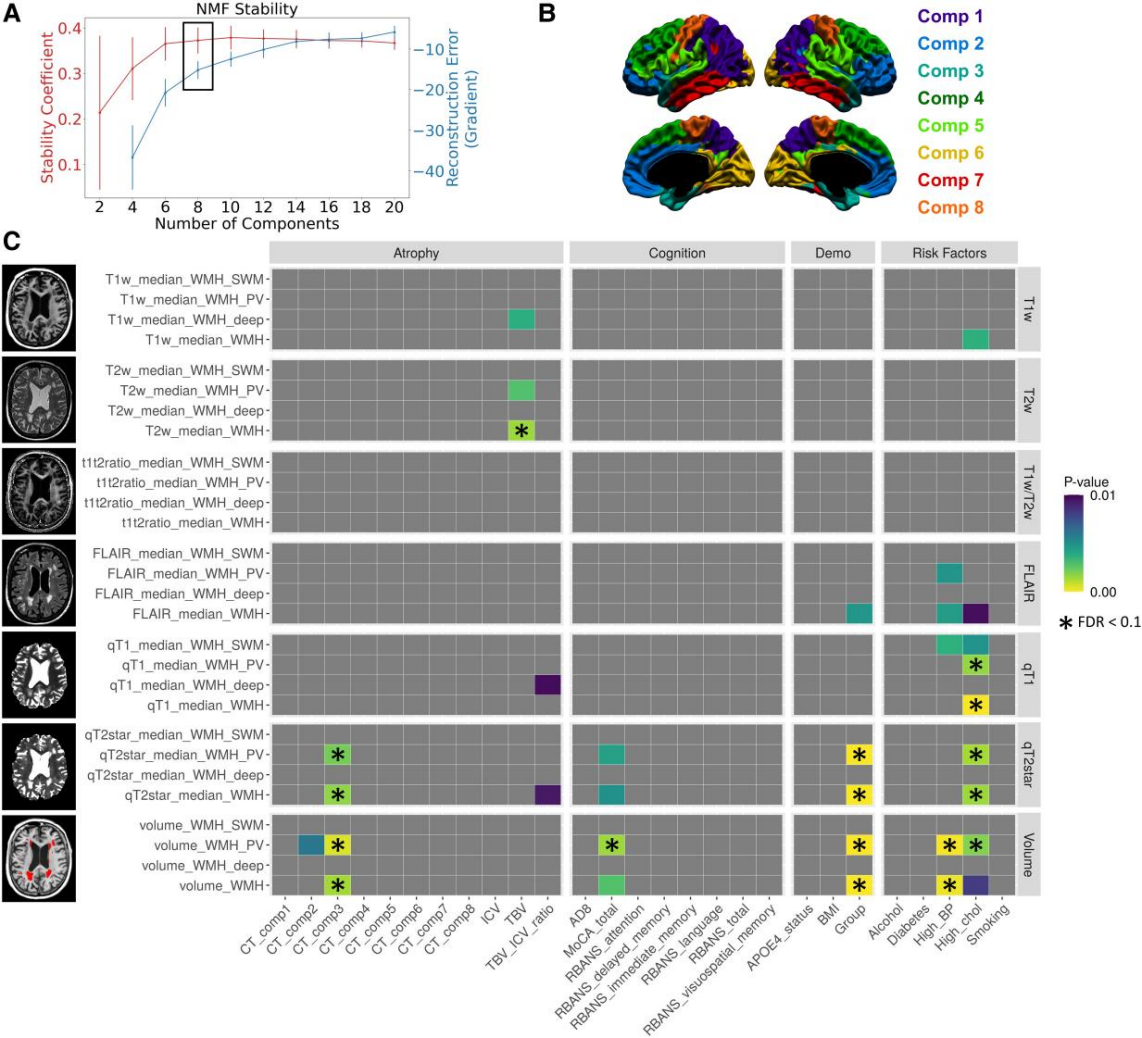
**Univariate analyses relating WMH characteristics to clinical variables (PV/deep/SWM parcellation).** (**A**) For each number of components of the NMF reconstruction, the stability (red) and the gradient of the reconstruction error (blue) are shown. The selected number of components is indicated by the black box. (**B**) Result of the winner-take-all NMF cortical thickness parcellation. Component 1 (purple) represents posterior temporo-parietal regions. Component 2 (blue) represents orbitofrontal, medial-frontal and anterior cingulate regions. Component 3 (turquoise) includes the medial temporal lobe and part of the temporal pole. Component 4 (dark green) represents posterior frontal regions. Component 5 (light green) represents the superior temporal gyrus and inferior parieto-frontal regions. Component 6 (yellow) represents the occipital lobes. Component 7 (red) represents the inferior and middle temporal gyri. Component 8 (orange) represents the sensorimotor cortex. (**C**) On the *y*-axis, all WMH measures (global and parcellated) are grouped by type of image. On the *x*-axis, all clinical variables are grouped by category. Associations between WMH signal measures and clinical variables were modelled with multiple linear regression for continuous measures and ANCOVAs for categorical measures. *P*-values of relationships between each WMH measure and each clinical variable (correcting for age, sex and education) are shown thresholded at *P* < 0.01, with non-significant associations in grey. Yellow colours indicate lower *P*-values, and purple colours indicate higher *P*-values. Relationships that survived FDR correction at the 0.1 level are indicated with a black star (*n* = 118).

Univariate relationships between WMH measures and different types of clinically-relevant variables were assessed, statistically controlling for age, sex and years of education. In the PV/deep/SWM parcellation (Fig. 4), we observed significant relationships for WMH volume in global and PV regions with atrophy (medial temporal lobe), cognition (MoCA), clinical group and cardiovascular risk factors (high BP and cholesterol). Similar relationships were observed for PV and global WMH qT2*, more specifically with atrophy (medial temporal lobe), cognition (MoCA, did not survive FDR correction), clinical group and cardiovascular risk factors (high cholesterol). While significant group effects were observed, there were no significant pairwise group differences (Supplementary Fig. 4). Other WMH measures did not show robust relationships across all types of clinical variables. When assessing the added value of WMH signal measures above the WMH volume (Supplementary Figs 6 and 7), qT2* remained significantly associated with atrophy (TBV/ICV ratio), clinical group and cardiovascular risk factors (high BP and high cholesterol), but not cognition.

In the lobar parcellation (Supplementary Figs 8 and 9), occipital WMH volume and qT2* were associated with a high number of atrophy, cognitive and risk factor variables, as well as group effects. WMH volume and qT2* in other lobar regions were also associated, although less extensively, with atrophy (medial temporal lobe), cognition (MoCA), clinical group and cardiovascular risk factors. Other WMH measures did not show robust relationships across all types of clinical variables.

When investigating standardized beta coefficients for continuous clinical predictors (Supplementary Figs 5, 7 and 9), the directionality of the associations for WMH qT2* and WMH volumes is in the expected direction (higher WMH volume and qT2* signal are related to lower cognition and cortical thickness).

### Multivariate relationships between brain and non-brain variables

**Figure 5:**
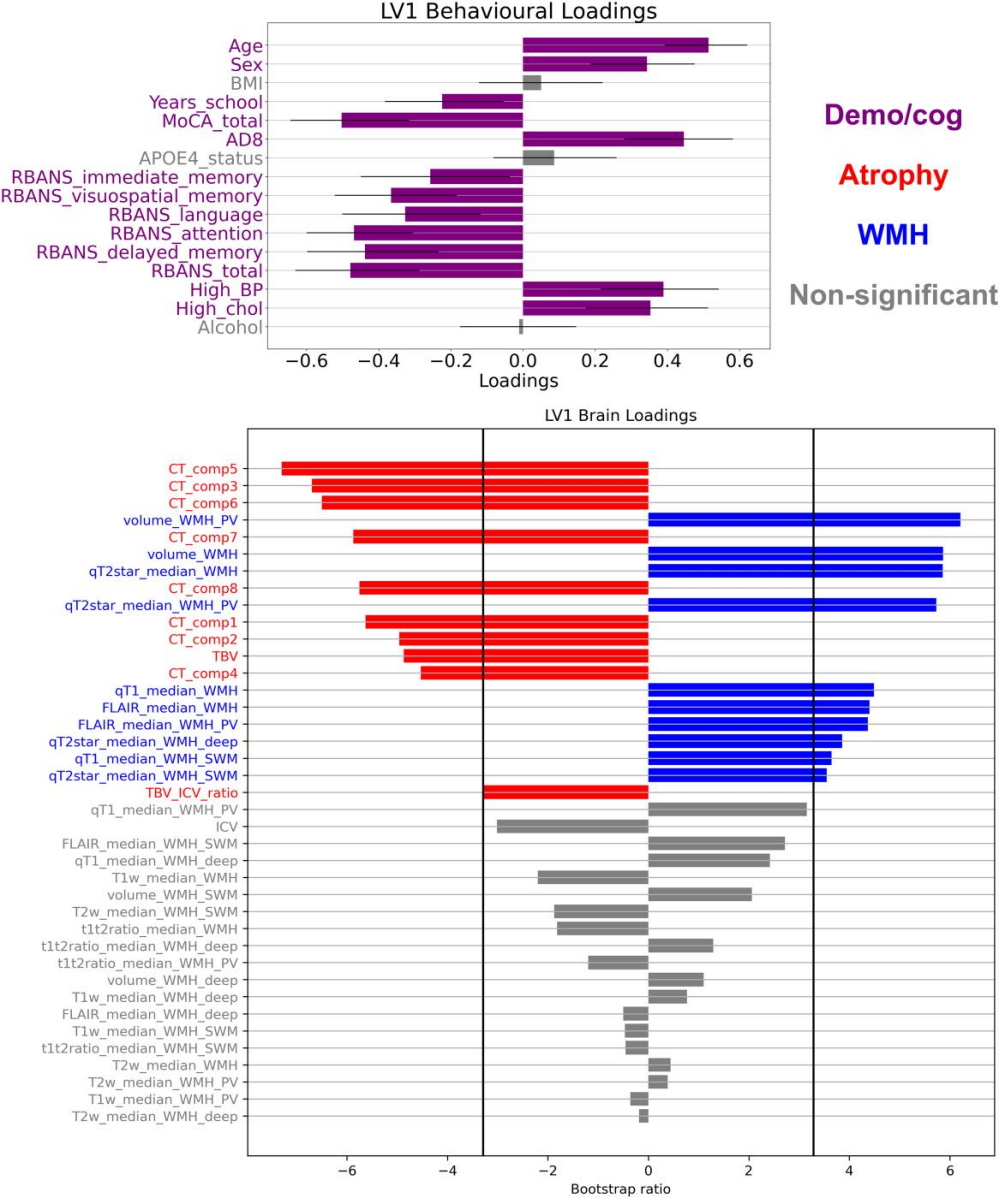
**Partial least squares correlation analysis relating brain variables to cognition and demographics (PV/deep/SWM parcellation).** Demographic and cognitive variables are shown in purple, atrophy variables in red, WMH variables in blue and non-significant variables in grey. *Top*: For each demographic and cognitive variable, the loading on LV1 is proportional to the correlation coefficient on the *x*-axis. 95% confidence intervals are shown, and variables contribute significantly to the LV (green) if the confidence interval does not cross 0. *Bottom*: For each brain variable, the bootstrap ratio (BSR) is proportional to the width of the bar on the *x*-axis. The variables are ordered from *top* to *bottom* by BSR value magnitude. Vertical lines at BSR ± 3.29 (equivalent to *P* < 0.001) indicate the significance thresholds (*n* = 118).

We further investigated multivariate relationships relating patterns of brain variables (WMH measures and cortical thickness) to patterns of cognitive and demographic variables with PLSC. In the PV/deep/SWM parcellation, LV1 explained the vast majority of the covariance between variables (83%), was significant (*P* = 0.0002) and survived split-half resampling (Supplementary Fig. 10A). LV3 was also significant (*P* = 0.007) and survived split-half resampling but only explained a very small proportion of the covariance (6%), thus is not further analysed but is available in Supplementary Fig. 10B. In LV1, a pattern of older age, female sex, lower education level, worst cognition (MoCA, AD8, RBANS) and risk factors (high blood pressure, high cholesterol) was related to a global pattern of lower cortical thickness and higher WMH volume and microstructural abnormality (Fig. 5). More specifically, the highest contributors to that pattern of brain variables according to the variable-specific bootstrap ratios were decreased cortical thickness in Components 5, 3 and 6 (respectively representing superior temporal regions, medial temporal lobe and occipital regions) and increased WMH volume and qT2* particularly in PV regions. Other WMH signal measures that were significant contributors at the bootstrap ratio > 3.29 level (equivalent to *P* < 0.001) were WMH qT2* signal in other white matter regions, qT1 signal in global and SWM WMHs and FLAIR signal in global and PV WMHs.

Similar results were obtained using the lobar parcellation (Supplementary Fig. 1^1^), with the first LV explaining the majority of the variance and showing a multivariate pattern of lower atrophy and higher WMH volume, qT2*, qT1 and FLAIR signal (especially in frontal regions) that is related to a pattern of older age, female sex, lower education level, worst cognition (MoCA, AD8, RBANS) and risk factors (high blood pressure, high cholesterol).

## Discussion

### qT2* as a potential indicator of tissue damage in WMHs

Our primary finding revealed the qT2* relaxation time of WMHs as a prime candidate for assessing WMH microstructural damage given the differential signal trends it demonstrated relative to NAWM, highlighting a sensitivity to tissue deterioration with age and vascular disease above what would be expected in the global white matter. We further observed consistent associations of WMH qT2* with cortical atrophy, clinical group differences, cardiovascular risk factors and to a lesser extent cognitive performance. Importantly, qT2* demonstrated predictive clinical value beyond WMH volume, highlighting the potentially complementary nature of these two measures to better characterize WMH severity. Given the known heterogeneity in the microstructural underpinnings of WMHs, qT2* could add crucial clinically-relevant and prognostic information when assessing the overall WMH burden.

Early in the process of WMH genesis, tissue alterations mostly represent accumulations of interstitial water content (i.e. oedema) due to disruptions in the blood–brain barrier.^63^ Following tissue damage includes inflammation,^64,65^ demyelination, axonal loss^15,19,66^ and death of oligodendrocytes.^67^ While a large portion of the qT2* signal can be attributed to variations in interstitial water content (positive association),^68^ studies demonstrated roughly equal contributions of iron (negative association) and myelin (negative association) to the qT2* signal in healthy white matter.^26^ Since most of the magnetic susceptibilities in white matter stems from paramagnetic iron, which is mostly localized in oligodendrocytes,^69^ a reduction in iron and magnetic susceptibilities likely indicates the death of oligodendrocytes. Integrating these known WMH microstructural alterations and sources of qT2* signal, we hypothesize that the increase of qT2* in WMHs is caused by a combination of increasing interstitial water content and decreasing myelin sheath and oligodendrocyte density. While qT2* might be less specific to any one of these microstructural properties compared to other quantitative MRI measures, it could be sensitive to a unique combination of these properties that best captures the overall severity of WMH tissue damage, thus resulting in the higher clinical associations we observed. Of note, the presence of microbleeds (another MRI-detectable consequence of small vessel disease) is likely not contributing to the qT2* effects we observed, even if they were located within WMHs, since they decrease the qT2* signal.

Only a limited number of studies have investigated T2-related properties of WMHs. Iordanishvili *et al.*^70^ observed a significant increase in PV qT2* with WMH volume, an effect that was not observed for NAWM, which supports our observations. One study used myelin water imaging, which separates the quantitative T2 signal into a short (myelin water sensitive) and long component (interstitial water sensitive), to investigate tissue alterations within WMHs.^71^ They observed that the myelin water fraction decreased with WMH volume and was lower in stroke populations, while the intra- and extra-cellular water fraction increased with WMH volume and was not different between stroke and normally-aging populations. Crucially, we extended this body of work by empirically demonstrating associations of the WMH qT2* signal with adverse neurobiological and clinical outcomes.

Interestingly, the clinical associations we observed were mostly for the qT2* signal in PV and occipital WMHs, suggesting that the location of damage is critical. PV WMHs have been more extensively associated with clinical outcomes but tend to represent a lower degree of demyelination compared to other WMHs.^6,16,17^ Similarly, studies show that the clinical impact of WMHs located in posterior regions (i.e. occipital and parietal lobes) is particularly significant in Alzheimer’s disease.^4,53,72^ As such, microstructural tissue damage of WMHs located in PV and occipital white matter regions, measured by the qT2* signal, could be particularly disruptive for cognitive processes and have a higher clinical impact. Alternatively, qT2* may be particularly sensitive to the tissue damage dynamics of WMHs in those regions.

### Limited clinical relevance of other signal measures of WMHs

Other signal measures of WMHs did not meet our criteria for clinical relevance since they did not show consistent associations with all categories of clinical variables and/or did not show divergent signal trends compared to NAWM.

We did not observe significant univariate relationships of WMH qT1 with cognition, atrophy and demographic variables, but we did observe significant univariate relationships with high cholesterol, as well as significant contributions of WMH qT1 to a multivariate pattern of age and cognition. qT1 in NAWM has been associated mostly with myelin (negative association) to a much larger extent than iron,^26^ and is highly influenced by interstitial water density (positive association).^73^ Interestingly, while the overall qT1 signal was different between WMH and NAWM, we observed that signal trends in aging and disease were highly similar, with signal in both regions slightly increasing with age and WMH volume. This can be explained by one of two possible mechanisms. First, although it is possible that the relationship between qT1 relaxation time and myelin does not hold in WMH tissue, Gouw *et al.*^18^ observed that the WMH qT1 signal was independently related to histologically-determined markers of myelin loss, but also axonal loss and microglial activation. The second possible explanation could imply the same rate of demyelination in WMHs and NAWM. Numerous studies have shown that myelin and qT1 alterations are widespread in the NAWM of patients with small vessel disease.^18,63,74,75^ We hypothesize that the initial increase in qT1 is due to oedema and that further qT1 increases might indicate a progressive loss of myelin sheaths that occurs at similar rates in WMH and NAWM. Nonetheless, we argue that, based on our results, qT1 is not a sensitive measure of microstructural tissue alterations specific to WMH.

While the WMH FLAIR signal showed highly divergent trends compared to NAWM, we observed few univariate relationships with clinical variables and some contributions to a multivariate pattern of aging and cognition. One study investigated microstructural substrates of FLAIR and T2w signal in WMHs and did not find associations between the intensity (i.e. brightness) of WMHs on these two images and the degree of axonal and myelin degradation.^66^ Since FLAIR, T1w, T2w and T1w/T2w ratio are qualitative MRI images and are thus more impacted by non-biological sources of variation, it is possible that this added noise compared to quantitative MRI limits the detectable associations with clinical variables and histopathological markers. Nevertheless, qT1 and FLAIR signals could potentially be of value in assessing the WMH microstructural damage given their multivariate relationships with clinical variables. Since FLAIR is not too highly correlated with qT2* signal (especially in PV regions), it could explain different sources of microstructural variations.

The clinical relevance of T1w, T2w and T1w/T2w ratio measures of WMH severity appears to be limited. Signal trends from all these measures were mostly not significantly different between WMH and NAWM, and we observed very few univariate and multivariate relationships with clinical variables. In contrast, some studies have used WMH T1w signal as a measure of WMH severity with the rationale that, since WMHs appear consistently smaller on this type of image, it could potentially be sensitive to more advanced tissue damage and demyelination.^76^ One study reported accelerated WMH T1w signal change after conversion from MCI to dementia, an effect that was not observed for WMH volume.^49^ This discrepancy with our findings could be due to differences in the samples: Dadar *et al.*^49^ used a longitudinal sample of 178 MCI subjects who converted to dementia, while our study was cross-sectional and only included 9 participants with dementia. As such, the T1w intensity could be especially sensitive to WMH severity in participants with more advanced neurodegeneration and small vessel disease. Still, our observations clearly show that other signal measures, such as qT2*, are more sensitive to WMH microstructural tissue damage.

### Clinical associations of increased WMH volumes

Our results largely recapitulate previously reported associations of WMH volume with clinical variables. Our observed associations between WMH volume and medial temporal lobe cortical thickness add to the growing body of literature suggesting that WMHs are associated with the stereotypical pattern of neurodegeneration in Alzheimer’s disease.^9,10,77^ Univariate relationships between WMH volume and cognition were less widespread than previously reported,^6,7^ as associations were restricted to MoCA scores and PV WMH volume, although additional relationships between occipital WMH volume and RBANS subscales (immediate memory, language and global cognition) were uncovered using a lobar parcellation. While we observed significant overall clinical group differences with respect to WMH volume, there were no significant pairwise group differences in the PV/deep/SWM parcellation. This is possibly due to a lack of power from the small number of participants with Alzheimer’s disease included in the final sample (*n* = 9), which possibly leads to an underestimation of the pathology level in our targeted population. Lastly, our observed associations between WMH volume and hypertension are consistent with the literature.^12,14^

### Separating WMHs located in deep and SWM regions

While the classical WMH parcellation only segregates periventricular and deep white matter regions, we differentiated WMHs located in the SWM, which we defined as 1 mm from the cortical grey matter. SWM is mostly composed of association U-fibres connecting cortical gyri and sulci and is thought to be relatively protected from vascular dysfunction since it is doubly vascularized,^78,79^ although WMHs located in SWM regions have been sparsely studied.^49^ However, we observed that there were more WMHs in SWM than in the deep white matter. This is possibly explained by the fact that the total SWM region was the largest on average, representing ∼47% of the total white matter in our parcellation scheme (Supplementary Fig. 3). Still, our results do not support reports of vascular protection of SWM since the majority of WMH voxels not in periventricular regions were within 1 mm of cortical grey matter.

### Strengths and limitations

The greatest strength of our study is our sample of participants across the Alzheimer’s disease spectrum, resulting in a higher degree of variability in WMH severity, neurodegeneration and cognitive functioning, thus increasing statistical power. Our sample is also well-suited for comparing signal measures of WMH since it included five different types of structural MRI acquisitions.

However, our study’s limitations include a somewhat limited sample size to detect associations between brain and clinical variables that are generally of small effect sizes, partly caused by a lack of phenotypical reliability of diagnostic and cognitive measures.^80,81^ This is why we used a stringent criterion to determine clinical relevance (i.e. consistent relationships across types of clinical variables, white matter parcellations and univariate and multivariate analyses). Despite the moderate sample size, qT2* clearly showed robust associations with neurobiological and clinical variables, and the fact that we observed highly similar clinical effects of WMH volumes with previous findings in the literature brings confidence to the generalizability of our results. Still, smaller associations of other WMH signal measures might not have been detected due to a lack of power.

One technical limitation is that no inhomogeneity corrections for quantitative images were used in this paper. While qT1 maps are not influenced by B1−, proton density, and T2* effects,^82^ several approaches to correct B1+ field inhomogeneities have been proposed^83,84^ but these require additional acquisitions, which we did not have access to. Additionally, while qT2* has been shown to be dependent on the orientation of white matter fibres with respect to the main magnetic field B0,^85,86^ no correction was used in the present paper. Furthermore, while it was necessary to normalize the intensities of weighted MRI images, our reference region (the genu of the corpus callosum) showed significant associations with WMH volume for the T1w/T2w ratio measure. This further highlights the advantage of quantitative MRI images in assessing tissue properties.

We did not include signal measures from diffusion tensor imaging (DTI), which are traditionally used to assess white matter integrity. Studies show that the DTI-derived microstructure within WMHs scales with WMH volume,^63,71^ and is associated with gait disturbances^87^ and cardiovascular risk factors.^88^ Reports have demonstrated that DTI alterations are detectable before the area appears hyperintense on FLAIR images, thus possibly being sensitive to early tissue alterations of WMHs, and show divergent longitudinal changes compared to NAWM.^89,90^ Hence, DTI metrics also show potential as signal measures of WMH tissue damage severity.

Lastly, it is not clear if our findings are generalizable outside of the Alzheimer’s disease spectrum. While some studies show generally similar microstructural substrates of WMHs between patients with and without Alzheimer’s disease,^15^ others report that some WMHs in Alzheimer’s disease are caused by cortical Alzheimer’s disease pathology through Wallerian degeneration and not small vessel disease, especially for WMHs located in parietal regions.^19^ However, our sample only contained a small number of subjects with Alzheimer’s disease (*n* = 9).

### Future directions

It will be important in future studies to assess the biological sources of the pathological increase of qT2* in WMHs, for example by relating the WMH qT2* signal to post-mortem histological markers of myelin and iron, as well as immunohistochemistry markers of ischaemia, blood–brain barrier dysfunction and inflammation. Future studies should also assess if the WMH qT2* signal is also clinically-relevant in different populations outside of the Alzheimer’s disease spectrum with high WMH prevalence, such as pure vascular dementia, psychiatric disorders and normal pressure hydrocephalus.^16^

An important potential use for assessing the degree of microstructural damage inside WMHs is to determine which WMHs represent irreversible tissue damage (i.e. myelin and axonal loss), or potentially reversible tissue damage (i.e. oedema). Indeed, a growing body of literature reports WMH volume reductions in some participants.^91^ Given that the rate of WMH volume change is associated with modifiable cardiovascular risk factors,^91^ it would be particularly relevant to identify if the WMHs of a patient could be reversible following cardiovascular interventions such as hypertensive medication, change in diet and increased physical activity. WMH signal measures, particularly qT2* but also possibly qT1 and FLAIR, could potentially add critical information in that regard, and as a result, could play a role in personalizing intervention strategies.

In summary, we assessed for the first time the clinical significance of qualitative and quantitative MRI WMH signal measures by relating them to cortical and global atrophy, cognition, clinical group along the Alzheimer’s disease spectrum and cardiovascular risk factors. We discovered that qT2* is a relevant marker of WMH microstructural damage, as it showed sensitivity to WMH-specific tissue degradation and was consistently associated with all types of clinical variables across different analysis schemes. qT1 and FLAIR could also be of interest, although to a lesser extent according to our results. We conclude that combining volumetric and signal measures of WMH should be used to improve the characterization of WMH severity *in vivo*.

## Supplementary Material

fcad279_Supplementary_Data

## Data Availability

All processing and analysis software used are free and open-access. The final data matrix, analysis code and results in raw form (*P*-values, coefficients, 95% confidence intervals) are available on our GitHub page (https://github.com/CoBrALab/WMH_Signal_AD_OParent_2023). The raw MRI data are available for research purposes from the corresponding author upon reasonable request.

## Appendix 1

List of PREVENT-AD collaborators

**Table:**

First Name	Initials	Last Name
Angela		Tam
Anne		Labonté
Alexa		Pichet Binette
Anne-Marie		Faubert
Axel		Mathieu
Cécile		Madjar
Charles Edouard		Carrier
Christian		Dansereau
Christina		Kazazian
Claude		Lepage
Cynthia		Picard
David		Maillet
Diane		Michaud
Doris		Couture
Doris		Dea
Claudio		Cuello
Alan		Barkun
Alan		Evans
Blandine		Courcot
Christine		Tardif
Clément		Debacker
Clifford	R.	Jack
David		Fontaine
David	S.	Knopman
Gerhard		Multhaup
Jamie		Near
Jeannie-Marie		Leoutsakos
Jean-Robert		Maltais
Jason		Brandt
Jens		Pruessner
John	C.	Morris
John	C.S.	Breitner
Judes		Poirier
Laksanun		Cheewakriengkrai
Lisa-Marie		MÃ¼nter
Louis		Collins
Mallar		Chakravarty
Mark	A.	Sager
Marina		Dauar-Tedeschi
Mark		Eisenberg
Natasha		Rajah
Paul		Aisen
Paule-Joanne		Toussaint
Pedro		Rosa-Neto
Pierre		Bellec
Penelope		Kostopoulos
Pierre		Etienne
Pierre	N.	Tariot
Pierre		Orban
Reisa	A.	Sperling
Rick		Hoge
Ronald	G.	Thomas
Serge		Gauthier
Suzanne		Craft
Sylvia		Villeneuve
Thomas	J.	Montine
Vasavan		Nair
Véronique		Bohbot
Vinod		Venugopalan
Vladimir		Fonov
Yasser		Ituria-Medina
Zaven	S.	Khachaturian
Eduard		Teigner
Elena		Anthal
Elsa		Yu
Fabiola		Ferdinand
Galina		Pogossova
Ginette		Mayrand
Guerda		Duclair
Guylaine		Gagné
Holly		Newbold-Fox
Illana		Leppert
Isabelle		Vallée
Jacob		Vogel
Jennifer		Tremblay-Mercier
Joanne		Frenette
Josée		Frappier
Justin		Kat
Justin		Miron
Karen		Wan
Laura		Mahar
Leopoldina		Carmo
Louise		Théroux
Mahsa		Dadar
Marianne		Dufour
Marie-Elyse		Lafaille-Magnan
Melissa		Appleby
Mélissa		Savard
Miranda		Tuwaig
Mirela		Petkova
Pierre		Rioux
Pierre-FranÃ§ois		Meyer
Rana		El-Khoury
Renee		Gordon
Renuka		Giles
Samir		Das
Seqian		Wang
Shirin		Tabrizi
Sulantha		Mathotaarachchi
Sylvie		Dubuc
Tanya		Lee
Thomas		Beaudry
Valérie		Gervais
Véronique		Pagé
Julie		Gonneaud
GÃ¼lebru		Ayranci
Tharick	A.	Pascoal
René		Desautels
Fatiha		Benbouhoud
Eunice Farah		Saint-Fort
Sander	C.J.	Verfaillie
Sarah		Farzin
Alyssa		Salaciak
Stephanie		Tullo
Etienne		Vachon-Presseau
Leslie-Ann		Daoust
Theresa		KÃ¶be
Nathan		Spreng
Melissa		McSweeney
Nathalie		Nilsson
Morteza		Pishnamazi
Christophe		Bedetti
Louise		Hudon
Claudia		Greco
Jean-Paul		Soucy

## References

[1] Kapasi A, DeCarli C, Schneider JA. Impact of multiple pathologies on the threshold for clinically overt dementia. Acta Neuropathol. 2017;134(2):171–186. 28488154 10.1007/s00401-017-1717-7 PMC5663642

[2] Bos D, Wolters FJ, Darweesh SKL, et al Cerebral small vessel disease and the risk of dementia: A systematic review and meta-analysis of population-based evidence. Alzheimers Dement. 2018;14(11):1482–1492. 29792871 10.1016/j.jalz.2018.04.007

[3] Iturria-Medina Y, Sotero RC, Toussaint PJ, et al Early role of vascular dysregulation on late-onset Alzheimer’s disease based on multifactorial data-driven analysis. Nat Commun. 2016;7(1):11934. 27327500 10.1038/ncomms11934 PMC4919512

[4] Lee S, Viqar F, Zimmerman ME, et al White matter hyperintensities are a core feature of Alzheimer’s disease: Evidence from the dominantly inherited Alzheimer network. Ann Neurol. 2016;79(6):929–939. 27016429 10.1002/ana.24647 PMC4884146

[5] Debette S, Markus HS. The clinical importance of white matter hyperintensities on brain magnetic resonance imaging: Systematic review and meta-analysis. BMJ. 2010;341:c3666. 20660506 10.1136/bmj.c3666 PMC2910261

[6] Roseborough AD, Saad L, Goodman M, Cipriano LE, Hachinski VC, Whitehead SN. White matter hyperintensities and longitudinal cognitive decline in cognitively normal populations and across diagnostic categories: A meta-analysis, systematic review, and recommendations for future study harmonization. Alzheimers Dement. 2022;19(1):194–207. 35319162 10.1002/alz.12642

[7] Kloppenborg RP, Nederkoorn PJ, Geerlings MI, van den Berg E. Presence and progression of white matter hyperintensities and cognition, a meta-analysis. Neurology. 2014;82(23):2127–2138. 24814849 10.1212/WNL.0000000000000505

[8] Dadar M, Manera AL, Ducharme S, Collins DL. White matter hyperintensities are associated with grey matter atrophy and cognitive decline in Alzheimer’s disease and frontotemporal dementia. Neurobiol Aging. 2022;111(March 2022):54–63. 34968832 10.1016/j.neurobiolaging.2021.11.007

[9] Habes M, Erus G, Toledo JB, et al White matter hyperintensities and imaging patterns of brain ageing in the general population. Brain. 2016;139(4):1164–1179. 26912649 10.1093/brain/aww008 PMC5006227

[10] Riphagen JM, Suresh MB, Salat DH. The canonical pattern of Alzheimer’s disease atrophy is linked to white matter hyperintensities in normal controls, differently in normal controls compared to in AD. Neurobiol Aging. 2022;114:105–112. 35414420 10.1016/j.neurobiolaging.2022.02.008 PMC9387174

[11] Rizvi B, Narkhede A, Last BS, et al The effect of white matter hyperintensities on cognition is mediated by cortical atrophy. Neurobiol Aging. 2018;64:25–32. 29328963 10.1016/j.neurobiolaging.2017.12.006 PMC5831564

[12] Abraham HMA, Wolfson L, Moscufo N, Guttmann CRG, Kaplan RF, White WB. Cardiovascular risk factors and small vessel disease of the brain: Blood pressure, white matter lesions, and functional decline in older persons. J Cereb Blood Flow Metab. 2016;36(1):132–142. 26036933 10.1038/jcbfm.2015.121 PMC4758547

[13] Debette S, Seshadri S, Beiser A, et al Midlife vascular risk factor exposure accelerates structural brain aging and cognitive decline. Neurology. 2011;77(5):461–468. 21810696 10.1212/WNL.0b013e318227b227 PMC3146307

[14] Newby D, Winchester L, Sproviero W, et al Associations between brain volumes and cognitive tests with hypertensive burden in UK Biobank. J Alzheimers Dis. 2021;84:1373–1389. 34690138 10.3233/JAD-210512 PMC8673518

[15] Gouw AA, Seewann A, Van Der Flier WM, et al Heterogeneity of small vessel disease: A systematic review of MRI and histopathology correlations. J Neurol Neurosurg Psychiatry. 2011;82(2):126–135. 20935330 10.1136/jnnp.2009.204685

[16] Kim KW, MacFall JR, Payne ME. Classification of white matter lesions on magnetic resonance imaging in elderly persons. Biol Psychiatry. 2008;64(4):273–280. 18471801 10.1016/j.biopsych.2008.03.024 PMC2593803

[17] Haller S, Kövari E, Herrmann FR, et al Do brain T2/FLAIR white matter hyperintensities correspond to myelin loss in normal aging? A radiologic–neuropathologic correlation study. Acta Neuropathol Commun. 2013;1(1):14. 24252608 10.1186/2051-5960-1-14 PMC3893472

[18] Gouw AA, Seewann A, Vrenken H, et al Heterogeneity of white matter hyperintensities in Alzheimer’s disease: Post-mortem quantitative MRI and neuropathology. Brain. 2008;131(12):3286–3298. 18927145 10.1093/brain/awn265

[19] McAleese KE, Walker L, Graham S, et al Parietal white matter lesions in Alzheimer’s disease are associated with cortical neurodegenerative pathology, but not with small vessel disease. Acta Neuropathol. 2017;134(3):459–473. 28638989 10.1007/s00401-017-1738-2 PMC5563333

[20] McAleese KE, Alafuzoff I, Charidimou A, et al Post-mortem assessment in vascular dementia: Advances and aspirations. BMC Med. 2016;14(1). 10.1186/s12916-016-0676-5 PMC5011905 27600683

[21] Glasser MF, Van Essen DC. Mapping human cortical areas in vivo based on myelin content as revealed by T1- and T2-weighted MRI. J Neurosci. 2011;31(32):11597–11616. 21832190 10.1523/JNEUROSCI.2180-11.2011 PMC3167149

[22] Parent O, Olafson E, Bussy A, et al High spatial overlap but diverging age-related trajectories of cortical magnetic resonance imaging markers aiming to represent intracortical myelin and microstructure. Hum Brain Mapp. 2023;44:3023–3044. 36896711 10.1002/hbm.26259 PMC10171508

[23] Tardif CL, Gauthier CJ, Steele CJ, et al Advanced MRI techniques to improve our understanding of experience-induced neuroplasticity. Neuroimage. 2016;131:55–72. 26318050 10.1016/j.neuroimage.2015.08.047

[24] Bahsoun MA, Khan MU, Mitha S, et al FLAIR MRI biomarkers of the normal appearing brain matter are related to cognition. NeuroImage Clin. 2022;34:102955. 35180579 10.1016/j.nicl.2022.102955 PMC8857609

[25] Deoni SCL . Quantitative relaxometry of the brain. Top Magn Reson Imaging. 2010;21(2):101–113. 21613875 10.1097/RMR.0b013e31821e56d8 PMC3613135

[26] Stüber C, Morawski M, Schäfer A, et al Myelin and iron concentration in the human brain: A quantitative study of MRI contrast. Neuroimage. 2014;93:95–106. 24607447 10.1016/j.neuroimage.2014.02.026

[27] Kor D, Birkl C, Ropele S, et al The role of iron and myelin in orientation dependent R2* of white matter. NMR Biomed. 2019;32:e4092. 31038240 10.1002/nbm.4092

[28] Bussy A, Patel R, Plitman E, et al Hippocampal shape across the healthy lifespan and its relationship with cognition. Neurobiol Aging. 2021;106:153–168. 34280848 10.1016/j.neurobiolaging.2021.03.018

[29] Bussy A, Plitman E, Patel R, et al Hippocampal subfield volumes across the healthy lifespan and the effects of MR sequence on estimates. Neuroimage. 2021;233:117931. 33677075 10.1016/j.neuroimage.2021.117931

[30] Tullo S, Patel R, Devenyi GA, et al MR-based age-related effects on the striatum, globus pallidus, and thalamus in healthy individuals across the adult lifespan. Hum Brain Mapp. 2019;40(18):5269–5288. 31452289 10.1002/hbm.24771 PMC6864890

[31] Breitner JCS, Poirier J, Etienne PE, Leoutsakos JM. Rationale and structure for a new center for studies on prevention of Alzheimer’s disease (StoP-AD). J Prev Alzheimers Dis. 2016;3(4):236–242. 29199324 10.14283/jpad.2016.121

[32] Tremblay-Mercier J, Madjar C, Das S, et al Open science datasets from PREVENT-AD, a longitudinal cohort of pre-symptomatic Alzheimer’s disease. Neuroimage Clin. 2021;31:102733. 34192666 10.1016/j.nicl.2021.102733 PMC8254111

[33] Lachman HM, Papolos DF, Saito T, Yu YM, Szumlanski CL, Weinshilboum RM. Human catechol-O-methyltransferase pharmacogenetics: Description of a functional polymorphism and its potential application to neuropsychiatric disorders. Pharmacogenetics. 1996;6(3):243–250. 8807664 10.1097/00008571-199606000-00007

[34] Galvin JE, Roe CM, Powlishta KK, et al The AD8: A brief informant interview to detect dementia. Neurology. 2005;65(4):559–564. 16116116 10.1212/01.wnl.0000172958.95282.2a

[35] Nasreddine ZS, Phillips NA, Bédirian V, et al The Montreal Cognitive Assessment, MoCA: A brief screening tool for mild cognitive impairment. J Am Geriatr Soc. 2005;53(4):695–699. 15817019 10.1111/j.1532-5415.2005.53221.x

[36] Randolph C, Tierney MC, Mohr E, Chase TN. The Repeatable Battery for the Assessment of Neuropsychological Status (RBANS): Preliminary clinical validity. J Clin Exp Neuropsychol. 1998;20(3):310–319. 9845158 10.1076/jcen.20.3.310.823

[37] Stekhoven DJ, Bühlmann P. MissForest—non-parametric missing value imputation for mixed-type data. Bioinformatics. 2012;28(1):112–118. 22039212 10.1093/bioinformatics/btr597

[38] Tustison NJ, Avants BB, Cook PA, et al N4ITK: Improved N3 bias correction. IEEE Trans Med Imaging. 2010;29(6):1310–1320. 20378467 10.1109/TMI.2010.2046908 PMC3071855

[39] Eskildsen SF, Coupé P, Fonov V, et al BEaST: Brain extraction based on nonlocal segmentation technique. Neuroimage. 2012;59(3):2362–2373. 21945694 10.1016/j.neuroimage.2011.09.012

[40] Kim JS, Singh V, Lee JK, et al Automated 3-D extraction and evaluation of the inner and outer cortical surfaces using a Laplacian map and partial volume effect classification. Neuroimage. 2005;27(1):210–221. 15896981 10.1016/j.neuroimage.2005.03.036

[41] Lerch JP, Evans AC. Cortical thickness analysis examined through power analysis and a population simulation. Neuroimage. 2005;24(1):163–173. 15588607 10.1016/j.neuroimage.2004.07.045

[42] Boucher M, Whitesides S, Evans A. Depth potential function for folding pattern representation, registration and analysis. Med Image Anal. 2009;13(2):203–214. 18996043 10.1016/j.media.2008.09.001

[43] Sotiras A, Resnick SM, Davatzikos C. Finding imaging patterns of structural covariance via non-negative matrix factorization. Neuroimage. 2015;108:1–16. 25497684 10.1016/j.neuroimage.2014.11.045 PMC4357179

[44] Patel R, Steele CJ, Chen AGX, et al Investigating microstructural variation in the human hippocampus using non-negative matrix factorization. Neuroimage. 2020;207:116348. 31715254 10.1016/j.neuroimage.2019.116348

[45] Robert C, Patel R, Blostein N, Steele CJ, Chakravarty MM. Analyses of microstructural variation in the human striatum using non-negative matrix factorization. Neuroimage. 2022;246:118744. 34848302 10.1016/j.neuroimage.2021.118744

[46] Avants BB, Tustison NJ, Song G, Cook PA, Klein A, Gee JC. A reproducible evaluation of ANTs similarity metric performance in brain image registration. Neuroimage. 2011;54(3):2033–2044. 20851191 10.1016/j.neuroimage.2010.09.025 PMC3065962

[47] Dadar M, Pascoal TA, Manitsirikul S, et al Validation of a regression technique for segmentation of white matter hyperintensities in Alzheimer’s disease. IEEE Trans Med Imaging. 2017;36(8):1758–1768. 28422655 10.1109/TMI.2017.2693978

[48] Dadar M, Maranzano J, Misquitta K, et al Performance comparison of 10 different classification techniques in segmenting white matter hyperintensities in aging. Neuroimage. 2017;157:233–249. 28602597 10.1016/j.neuroimage.2017.06.009 PMC6469398

[49] Dadar M, Maranzano J, Ducharme S, Collins DL. White matter in different regions evolves differently during progression to dementia. Neurobiol Aging. 2019;76:71–79. 30703628 10.1016/j.neurobiolaging.2018.12.004

[50] DeCarli C, Fletcher E, Ramey V, Harvey D, Jagust WJ. Anatomical mapping of white matter hyperintensities (WMH): Exploring the relationships between periventricular WMH, deep WMH, and total WMH burden. Stroke. 2005;36(1):50–55. 15576652 10.1161/01.STR.0000150668.58689.f2 PMC3816357

[51] Griffanti L, Jenkinson M, Suri S, et al Classification and characterization of periventricular and deep white matter hyperintensities on MRI: A study in older adults. Neuroimage. 2018;170:174–181. 28315460 10.1016/j.neuroimage.2017.03.024

[52] Huang CC, Yang AC, Chou KH, et al Nonlinear pattern of the emergence of white matter hyperintensity in healthy Han Chinese: An adult lifespan study. Neurobiol Aging. 2018;67:99–107. 29655051 10.1016/j.neurobiolaging.2018.03.012

[53] Brickman AM, Zahodne LB, Guzman VA, et al Reconsidering harbingers of dementia: Progression of parietal lobe white matter hyperintensities predicts Alzheimer’s disease incidence. Neurobiol Aging. 2015;36(1):27–32. 25155654 10.1016/j.neurobiolaging.2014.07.019 PMC4268124

[54] Dadar M, Maranzano J, Ducharme S, Carmichael OT, Decarli C, Collins DL. Validation of T1w-based segmentations of white matter hyperintensity volumes in large-scale datasets of aging. Hum Brain Mapp. 2018;39(3):1093–1107. 29181872 10.1002/hbm.23894 PMC6866430

[55] Hammers A, Allom R, Koepp MJ, et al Three-dimensional maximum probability atlas of the human brain, with particular reference to the temporal lobe. Hum Brain Mapp. 2003;19(4):224–247. 12874777 10.1002/hbm.10123 PMC6871794

[56] Dadar M, Mahmoud S, Zhernovaia M, Camicioli R, Maranzano J, Duchesne S. White matter hyperintensity distribution differences in aging and neurodegenerative disease cohorts. NeuroImage: Clinical. 2022;36:103204. 36155321 10.1016/j.nicl.2022.103204 PMC9668605

[57] Andere A, Jindal G, Molino J, et al Volumetric white matter hyperintensity ranges correspond to Fazekas scores on brain MRI. J Stroke Cerebrovasc Dis. 2022;31(4):106333. 35158149 10.1016/j.jstrokecerebrovasdis.2022.106333

[58] Genovese CR, Lazar NA, Nichols T. Thresholding of statistical maps in functional neuroimaging using the false discovery rate. Neuroimage. 2002;15(4):870–878. 11906227 10.1006/nimg.2001.1037

[59] Krishnan A, Williams LJ, McIntosh AR, Abdi H. Partial Least Squares (PLS) methods for neuroimaging: A tutorial and review. Neuroimage. 2011;56(2):455–475. 20656037 10.1016/j.neuroimage.2010.07.034

[60] McIntosh AR, Mišić B. Multivariate statistical analyses for neuroimaging data. Annu Rev Psychol. 2013;64(1):499–525. 22804773 10.1146/annurev-psych-113011-143804

[61] Zeighami Y, Fereshtehnejad SM, Dadar M, et al A clinical-anatomical signature of Parkinson’s disease identified with partial least squares and magnetic resonance imaging. Neuroimage. 2019;190:69–78. 29277406 10.1016/j.neuroimage.2017.12.050

[62] Hansen JY, Markello RD, Vogel JW, Seidlitz J, Bzdok D, Misic B. Molecular signatures of cognition and affect. *bioRxiv*.

[63] Muñoz Maniega S, Chappell FM, Valdés Hernández MC, et al Integrity of normal-appearing white matter: Influence of age, visible lesion burden and hypertension in patients with small-vessel disease. J Cereb Blood Flow Metab. 2017;37(2):644–656. 26933133 10.1177/0271678X16635657 PMC5381455

[64] Simpson JE, Fernando MS, Clark L, et al White matter lesions in an unselected cohort of the elderly: Astrocytic, microglial and oligodendrocyte precursor cell responses. Neuropathol Appl Neurobiol. 2007;33(4):410–419. 17442062 10.1111/j.1365-2990.2007.00828.x

[65] Swardfager W, Yu D, Ramirez J, et al Peripheral inflammatory markers indicate microstructural damage within periventricular white matter hyperintensities in Alzheimer’s disease: A preliminary report. Alzheimers Dement. 2017;7:56–60. 10.1016/j.dadm.2016.12.011 PMC5328682 28275700

[66] Roseborough AD, Langdon KD, Hammond R, et al Post-mortem 7 Tesla MRI detection of white matter hyperintensities: A multidisciplinary voxel-wise comparison of imaging and histological correlates. NeuroImage: Clinical. 2020;27:102340. 32679554 10.1016/j.nicl.2020.102340 PMC7364158

[67] Rajani RM, Quick S, Ruigrok SR, et al Reversal of endothelial dysfunction reduces white matter vulnerability in cerebral small vessel disease in rats. Sci Transl Med. 2018;10(448):eaam9507. 29973407 10.1126/scitranslmed.aam9507

[68] Lee J, Hyun JW, Lee J, et al So you want to image myelin using MRI: An overview and practical guide for myelin water imaging. J Magn Reson Imaging. 2021;53(2):360–373. 32009271 10.1002/jmri.27059

[69] Todorich B, Pasquini JM, Garcia CI, Paez PM, Connor JR. Oligodendrocytes and myelination: The role of iron. Glia. 2009;57(5):467–478. 18837051 10.1002/glia.20784

[70] Iordanishvili E, Schall M, Loução R, et al Quantitative MRI of cerebral white matter hyperintensities: A new approach towards understanding the underlying pathology. Neuroimage. 2019;202:116077. 31398433 10.1016/j.neuroimage.2019.116077

[71] Ferris JK, Greeley B, Vavasour IM, et al In vivo myelin imaging and tissue microstructure in white matter hyperintensities and perilesional white matter. Brain Commun. 2022;4(3):fcac142. 35694147 10.1093/braincomms/fcac142 PMC9178967

[72] Skrobot OA, Attems J, Esiri M, et al Vascular cognitive impairment neuropathology guidelines (VCING): The contribution of cerebrovascular pathology to cognitive impairment. Brain. 2016;139(11):2957–2969. 27591113 10.1093/brain/aww214

[73] Naruse S, Horikawa Y, Tanaka C, Hirakawa K, Nishikawa H, Yoshizaki K. Significance of proton relaxation time measurement in brain edema, cerebral infarction and brain tumors. Magn Reson Imaging. 1986;4(4):293–304. 3669944 10.1016/0730-725x(86)91039-8

[74] Park M, Moon Y, Han SH, Kim HK, Moon WJ. Myelin loss in white matter hyperintensities and normal-appearing white matter of cognitively impaired patients: A quantitative synthetic magnetic resonance imaging study. Eur Radiol. 2019;29(9):4914–4921. 30488109 10.1007/s00330-018-5836-x

[75] Maniega Muñoz S, Valdés Hernández MC, Clayden JD, et al White matter hyperintensities and normal-appearing white matter integrity in the aging brain. Neurobiol Aging. 2015;36(2):909–918. 25457555 10.1016/j.neurobiolaging.2014.07.048 PMC4321830

[76] Melazzini L, Mackay CE, Bordin V, et al White matter hyperintensities classified according to intensity and spatial location reveal specific associations with cognitive performance. Neuroimage Clin. 2021;30:102616. 33743476 10.1016/j.nicl.2021.102616 PMC7995650

[77] Appel J, Potter E, Bhatia N, et al Association of white matter hyperintensity measurements on brain MR imaging with cognitive status, medial temporal atrophy, and cardiovascular risk factors. AJNR Am J Neuroradiol. 2009;30(10):1870–1876. 19643919 10.3174/ajnr.A1693 PMC7051285

[78] Smirnov M, Destrieux C, Maldonado IL. Cerebral white matter vasculature: Still uncharted? Brain. 2021;144(12):3561–3575. 34718425 10.1093/brain/awab273

[79] Moody DM, Bell MA, Challa VR. Features of the cerebral vascular pattern that predict vulnerability to perfusion or oxygenation deficiency: An anatomic study. AJNR Am J Neuroradiol. 1990;11(3):431–439. 2112304 PMC8367475

[80] Marek S, Tervo-Clemmens B, Calabro FJ, et al Reproducible brain-wide association studies require thousands of individuals. Nature. 2022;603:654–660. 35296861 10.1038/s41586-022-04492-9 PMC8991999

[81] Nikolaidis A, Chen AA, He X, et al Suboptimal phenotypic reliability impedes reproducible human neuroscience. *bioRxiv*.

[82] Marques JP, Kober T, Krueger G, van der Zwaag W, Van de Moortele PF, Gruetter R. MP2RAGE, a self bias-field corrected sequence for improved segmentation and T1-mapping at high field. Neuroimage. 2010;49(2):1271–1281. 19819338 10.1016/j.neuroimage.2009.10.002

[83] Lutti A, Hutton C, Finsterbusch J, Helms G, Weiskopf N. Optimization and validation of methods for mapping of the radiofrequency transmit field at 3T. Magn Reson Med. 2010;64(1):229–238. 20572153 10.1002/mrm.22421 PMC3077518

[84] Weiskopf N, Lutti A, Helms G, Novak M, Ashburner J, Hutton C. Unified segmentation based correction of R1 brain maps for RF transmit field inhomogeneities (UNICORT). Neuroimage. 2011;54(3):2116–2124. 20965260 10.1016/j.neuroimage.2010.10.023 PMC3018573

[85] Cherubini A, Péran P, Hagberg G, Caltagirone C, Sabatini U, Spalletta G. Characterization of white matter fiber bundles with T2* relaxometry and DTI. NeuroImage. 2009;47:S105. 10.1002/mrm.21978 19253372

[86] Bender B, Klose U. The in vivo influence of white matter fiber orientation towards B(0) on T2* in the human brain. NMR Biomed. 2010;23(9):1071–1076. 20665897 10.1002/nbm.1534

[87] de Laat KF, van Norden AGW, Gons RAR, et al Diffusion tensor imaging and gait in elderly persons with cerebral small vessel disease. Stroke. 2011;42(2):373–379. 21193751 10.1161/STROKEAHA.110.596502

[88] Mayer C, Nägele FL, Petersen M, et al Free-water diffusion MRI detects structural alterations surrounding white matter hyperintensities in the early stage of cerebral small vessel disease. J Cereb Blood Flow Metab. 2022;42(9):1707–1718. 35410517 10.1177/0271678X221093579 PMC9441727

[89] Van Leijsen EMC, Bergkamp MI, Van Uden IWM, et al Progression of white matter hyperintensities preceded by heterogeneous decline of microstructural integrity. Stroke. 2018;49(6):1386–1393. 29724890 10.1161/STROKEAHA.118.020980

[90] de Groot M, Verhaaren BFJ, de Boer R, et al Changes in normal-appearing white matter precede development of white matter lesions. Stroke. 2013;44(4):1037–1042. 23429507 10.1161/STROKEAHA.112.680223

[91] van Leijsen EMC, de Leeuw FE, Tuladhar AM. Disease progression and regression in sporadic small vessel disease-insights from neuroimaging. Clin Sci. 2017;131(12):1191–1206. 10.1042/CS20160384 28566448

